# Does catheter material affect functional performance of intravenous ports via the superior vena cava?

**DOI:** 10.1371/journal.pone.0253818

**Published:** 2021-10-27

**Authors:** Ching-Feng Wu, Jui-Ying Fu, Tsai-Yang Hsieh, Chi-Tsung Wen, Sheng-Yueh Yu, Ming-Ju Hsieh, Yen Chu, Ching-Yang Wu

**Affiliations:** 1 Division of Thoracic and Cardiovascular Surgery, Department of Surgery, Chang Gung Memorial Hospital, Linkou, Taiwan; 2 College of Medicine, Chang Gung University, Taoyuan, Taiwan; 3 Division of Critical Care and Pulmonary Medicine, Department of Internal Medicine, Chang Gung Memorial Hospital, Linkou, Taiwan; 4 Division of Thoracic and Cardiovascular Surgery, Department of Surgery, New Taipei Municipal Tu-Cheng Hospital, New Taipei City, Taiwan; 5 Laboratory of Cardiovascular Physiology, Department of Medical Research and Development, Chang Gung Memorial Hospital, Linkou, Taiwan; Ohio State University Wexner Medical Center Department of Surgery, UNITED STATES

## Abstract

**Introduction:**

The catheter is the only intravascular portion of an implanted port and plays a crucial role in catheter related complications. Both polyurethane and silicone are biocompatible materials which are utilized for catheter manufacturing, but their correlation to complications remains controversial. The aim of this study was to try to analyze the relationship between catheter materials and complications.

**Materials and methods:**

A total of 3144 patients who underwent intravenous port implantation between March 2012 and December 2018 at Chang Gung Memorial Hospital, Linkou, Taiwan were recruited. Of these, 1226 patients received silicone catheter port implantation and 1679 received polyurethane catheter ports. Case matching was done prior to analysis and catheter related complications and cumulative complication incidence for each group were compared.

**Results:**

Intergroup differences were identified in entry vessel (p = 0.0441), operation year (p < 0.0001), operation method (p = 0.0095), functional period (p < 0.0001), patient follow up status (p < 0.0001), operating time for vessel cutdown (p < 0.0001) and wire assisted approach (p = 0.0008). Stratified by specific entry vessel, no statistical difference was found in complication rate or incidence between the silicone and polyurethane groups. We further compared the cumulative complication incidence of the silicone and polyurethane groups, and also found no statistical difference (p = 0.4451).

**Conclusion:**

As long as external stress forces generated by surrounding structures and focused on potential weak points are avoided, both silicone and polyurethane materials provide sufficient structural stability to serve as reliable vascular access for patients.

## Introduction

Intravenous ports available for clinical use are composed of three components, including injection chamber, locking nut, and catheter. The catheter is the only component that really resides within the vessel and plays a crucial role in catheter related complications. It can be made of silicone or polyurethane since both are biochemically compatible materials that have been approved for long term implantation. Polyurethane produced for hard segments consists of linear, aromatic or aliphatic polyurethane chains [1], while that for soft segments consists of linear aliphatic polyether, polyester or polycarbonate chains. The irregular crystalline and amorphous structure of hard and soft segment types creates the highly elastomeric characteristics of the material. Silicone is a polymer consisting of a silicon-oxygen backbone with hydrocarbon side groups. The hydrocarbon side chains are additionally cross-linked with peroxides, resulting in elastomeric characteristics. As a result of the molecular difference, polyurethane is tougher and has five times the tensile strength of silicone [1]. This allows polyurethane catheters to have a thinner wall and larger intraluminal caliber, resulting in higher flow, compared to silicone catheters with the same outer caliber [2,3]. Both materials suffered degradation and weakening in strength in an ex vivo simulation [4–6], implying catastrophic complications if the materials fail in a real world setting.

In clinical practice, intravenous ports are implanted by either vessel cutdown [7–9], blind puncture [10,11], or echo guided puncture [12,13]. From the literature review, the reported early and late complication rates range from 0 to 1.8% and 9.1 to 21.06%, respectively [14]. Most studies have pooled all implanted chest ports manufactured by different companies and focused on complications, but have not further analyzed the effect of material differences. A few studies have tried to analyze the effect of different materials but their results have failed to generate a consensus [15–17]. Many clinical factors, such as implantation method, quality of implantation, patients’ body characteristics, and underlying malignancy may affect the function of ports. From the view of implantation method, catheters implanted by subclavian vein puncture wound are subject to external repetitive compression generated from the 1st rib and clavicle, leading to material fatigue and resulting in pinch-off syndrome [18]. In addition, the quality of implantation is also crucial for the long-term functioning of the port. An implanted port should have a smooth contour and appropriate tip location [14]. Inadequate pocket creation can lead to catheter impingement at the junction site between the catheter and locking nut, and can be identified in post-operation chest plain film by the small nut-catheter angle. The stress generated by the surrounding tissue becomes focused at this site, resulting in material failure [19]. The impingement also results in compromised catheter lumen and can lead to inadequate maintenance and subsequent malfunction [20]. Tip location is also crucial for a functional port since shallow catheter tip location has been correlated to migration and malfunction [20,21]. From the view of patients’ body characteristics, obese male patients and females with heavy breasts have abundant subcutaneous adipose tissue that can push the port upward while lying down, resulting in catheter impingement at the junction site between the locking nut and proximal catheter [19]. This leads to catheter compression which may develop into catheter malfunction or fracture. Patients’ particular type of malignancy, chemotherapeutic agent and patients’ survival may also affect the long-term function of implanted ports. These confounding factors must be corrected for, prior to analyzing the effect of different catheter materials. The aim of this study was to try to identify the correlation between catheter material and catheter related complications after correcting for these clinical confounding factors.

## Materials and methods

### Patient population

**Fig 1:**
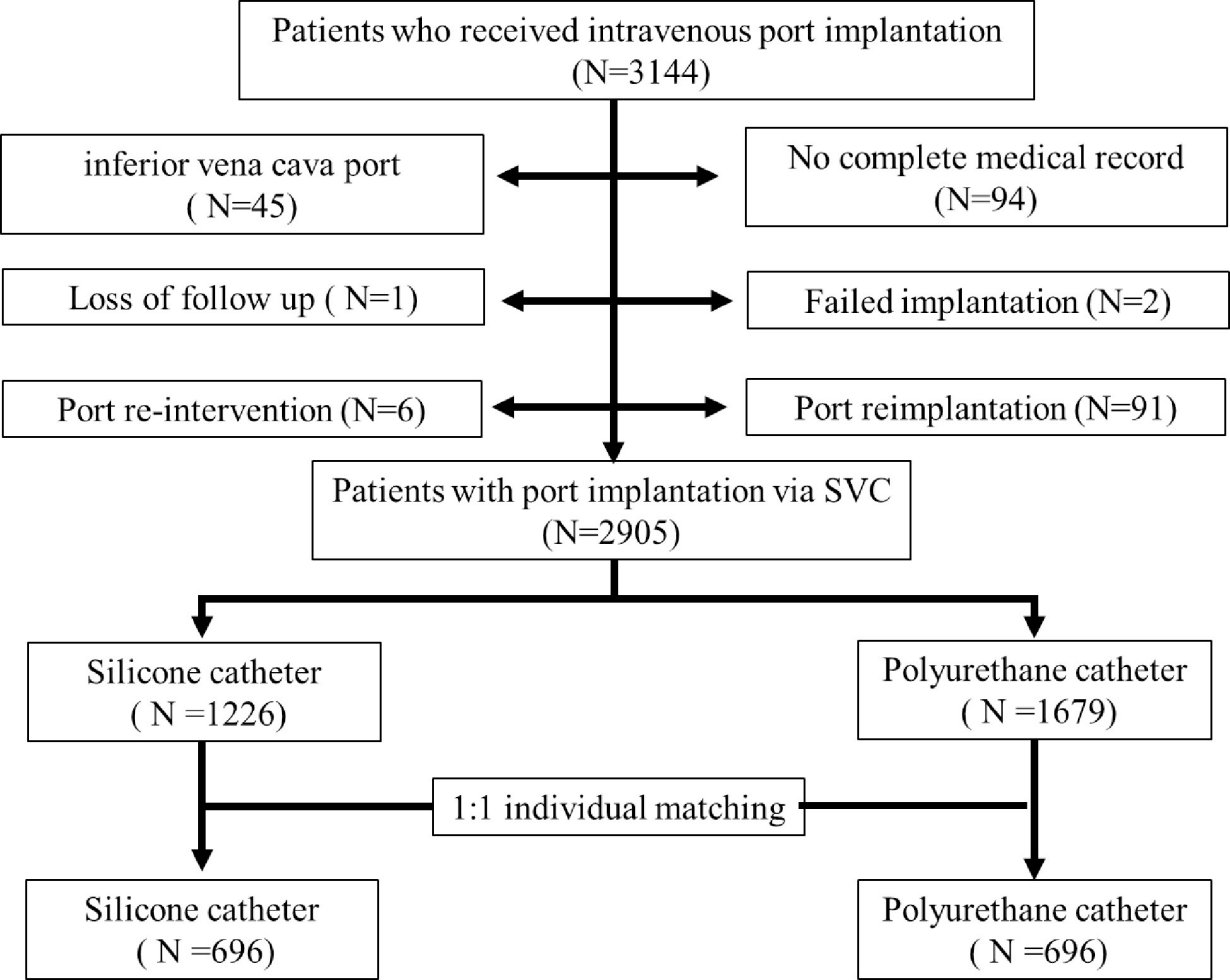
Patients’ disposition diagram. Paired variables: Gender, age±1, BMI±1, Underlying malignancy (Head and neck, Thorax, Abdomen, Pelvis, Soft tissue, Hematology and Other).

A total of 3144 patients who underwent intravenous port implantation between March 2012 and December 2018 at Chang Gung Memorial Hospital, Linkou, Taiwan were recruited. Patients who received port implantation via the inferior vena cava (IVC), received port re-intervention or re-implantation, were disqualified from implantation, were lost to follow up or were without complete medical records were excluded. A total of 2905 patients who underwent port implantation via the superior vena cava (SVC) route were included (Fig 1). Of these, 1226 patients who received silicone catheter port implantation were designated as the silicone group, while 1679 patients who received a polyurethane catheter port were designated as the polyurethane group. Informed consent was waived and this retrospective study was approved by the Institutional Review Board under the number 201800329B0.

### Decision algorithm and operation procedure for implantation

Patients’ tumor status, further treatment planning, the embedding pocket sites and vessel patency were taken into consideration prior to operation [14,22]. The intravenous port was implanted in an area with adequate overlying flaps and far from other treatments. The procedure was done in an area near an anatomic landmark, eg. the coracoid process of the scapulae, under local anesthesia after aseptic preparation. A sub-clavicular incision of 2 cm was made and deepened to the delto-pectoral groove. The entry vessel was chosen by the following order of priority: cephalic vein, deltoid branch of the thoraco-acromial vein, and internal jugular vein (IJV), whereby another vessel, such as the axillary vein was selected if the first three options were unavailable. Vessel cutdown was the preferred implantation method. Metallic wire assistance with or without puncture was reserved for patients with inadequate vessel caliber or tortuous route. For those where wire cannulation proved difficult, intra-operation venography was done to clarify the cannulation path and establish the implantation route prior to tunnel creation by puncture sheath. The implanted catheter length was determined according to the patient’s body height and location of the carina [14,22]. Intraoperative fluoroscopy was employed for implantation guidance and catheter tip confirmation.

### Implanted device and maintenance protocol

Four different types of implanted ports, including Celsite® (B. Braun Medical, Saint-Cloud, France), Polysite® (Perouse Medical, Ivry le Temple, France), Bard X port (Beckton Dickson and Company, New Jersey, United States), and Bard Power Port (Beckton Dickson and Company, New Jersey, United States) were implanted during the study period. After therapeutic use, the implanted ports underwent irrigation with 10 ml 0.9% normal saline, followed by heparin lock (50 IU/ ml). In addition, the implanted ports were maintained by irrigation at 3-month intervals after chemotherapy was completed.

### Definition of catheter-related complications

The definition of infection was if fever was noted during irrigation or if blood culture sampled from the port was positive for microorganism growth. Catheter malfunction was defined as a catheter that could not be smoothly pushed. Catheter tip migration was defined as a tip having migrated to a vessel from the SVC and RA junction. Port rotation was defined as a port body having turned upside down so that the silicone diaphragm could not be accessed for puncture. Deep vein thrombosis was defined as ipsilateral upper arm swelling and peripheral vessel confirmed ipsilateral subclavian thrombosis.

### Postoperative surveillance and follow-up

Postoperative chest plain films were done to clarify integrity and catheter route and to assess catheter-nut angle and catheter tip. Catheter-nut angle and tip location were measured by picture archiving and communication system (PACS). The functional period of implanted ports for patients with and without complications was defined as the interval between implantation and re-intervention and between implantation and last follow-up, respectively. All patients returned to the outpatient department for follow-up at 3-month intervals and underwent flushing for maintenance.

### Statistics

All collected data were first analyzed using univariate analysis. Categorical variables were compared using chi-square test or Fisher’s exact test. A p-value of less than 0.05 is considered statistically significant, and confidence intervals (CI) are assumed to have a coverage probability of 95%. Complication rates are presented as episode percentage among the whole population and incidences are presented as episodes per 1000 catheter days. Patients in the silicone and polyurethane group were matched according to patients’ characteristics, including gender, age, body mass index (BMI) and underlying malignancy prior to analysis. Competing risk was evaluated to analyze the impact of different catheter materials on the functional periods of implanted ports [23]. All analyses were performed using SAS, version 9 (SAS Institute, North Carolina, USA).

## Results

Before matching, 1226 patients who received silicone catheter port implantations and 1679 patients who received polyurethane catheter implantations were identified (S1 Table). In order to minimize confounding by patients’ characteristics, including gender, age, body mass index (BMI) and underlying malignancy, patients were matched prior to further analysis (Fig 1). After patient matching, both groups had 696 patients and the descriptive data is shown in Table 1. There were no statistical differences in gender (p = 1.0000), age (59.55 ± 11.26 vs. 59.55 ± 11.25 years, p = 0.9943), BMI (23.01 ± 3.21vs. 23.03 ± 3.20 kg/ m2, p = 0.9174), underlying malignancy (p = 1.0000), implantation side (p = 0.1616), nut-catheter angle (169.86° ± 7.15° vs. 169.87° ± 7.60°, p = 0.9887) and catheter tip location (1.06 ± 1.30 vs. 1.18 ± 1.45 cm, p = 0.1059) between the silicone and the polyurethane group (Table 1). Statistical differences between the groups were identified in entry vessel (p = 0.0441), operation year (p < 0.0001), operation method (p = 0.0095), functional period (667.90 ± 589.66 vs. 518.00 ± 452.81 days, p< 0.0001), patient follow up status (p< 0.0001), operation time in vessel cutdown (29.23 ± 9.42 vs. 24.57 ± 9.27 min, p< 0.0001) and wire assisted approach time (32.67 ± 9.76 vs. 28.46 ± 11.38 min, p = 0.0008).

**Table 1:** Descriptive data of ports with silicone and polyurethane catheters (after matching).

Group Variables	Silicone (n = 696) N(%)/ mean±SD	Polyurethane (n = 696) N (%)/ mean±SD	p-value^1^	Group Variables	Silicone (n = 696) N(%)/mean± SD	Polyurethane (n = 696) N (%)/ mean± SD	p-value^1^
Gender Female Male	276 (39.66%) 420 (60.34%)	276 (39.66%) 420 (60.34%)	1.000	Operation method Vessel cutdown Wire assistance without puncture Wire assistance with puncture Wire assistance with venogram a. Over the wire b. Modified puncture Echo guide puncture	428 (61.49%) 156 (22.41%) 75 (10.78%) 3 (0.43%) 2 (0.29%) 32 (4.60%)	439 (63.07%) 136 (19.54%) 88 (12.64%) 3 (0.43%) 13 (1.87%) 17 (2.44%)	0.0095
Age	59.55 ± 11.26	59.55 ± 11.25	0.9943				
Body height	160.85 ± 8.29	161.73 ± 8.50	0.0509				
Body weight	59.67 ± 10.22	60.34 ± 10.15	0.2193				
Body mass index	23.01 ± 3.21	23.03 ± 3.20	0.9174				
Malignancy Head and neck Thorax Abdomen Pelvis Soft tissue Hematology Other	57 (8.19%) 335 (48.13%) 269 (38.65%) 1 (0.14%) 0 (0.00%) 34 (4.89%) 1 (0.14%)	57 (8.19%) 335 (48.13%) 269 (38.65%) 1 (0.14%) 0 (0.00%) 34 (4.89%) 1 (0.14%)	1.0000 1.0000 1.0000 1.0000 N/A 1.0000 1.0000				
				Operation time Vessel cutdown Wire assistance without puncture Wire assistance with puncture Wire assistance with venogram a. Over the wire b. Modified puncture Echo guide puncture	29.23 ± 9.42 32.67 ± 9.76 44.83 ± 16.59 27.67 ± 10.97 44.00 ± 1.41 62.25 ± 22.36	24.57 ± 9.27 28.46 ± 11.38 40.68 ± 17.07 30.33 ± 10.97 46.92 ± 13.17 59.24 ± 13.29	<0.0001 0.0008 0.1195 0.7807 0.7660 0.5574
Side Right Left	631 (90.66%) 65 (9.34%)	615 (88.36%) 81 (11.64%)	0.1616				
				Port type B’Braun Fr. 6.5 (Silicone) Polysite Fr.7 (Silicone) Bard Power port Fr.6 (Polyurethane) Bard Fr. port 6/8 X (Polyurethane)	511 (73.42%) 185 (26.58%) 0 (0.00%) 0 (0.00%)	0 (0.00%) 0 (0.00%) 363 (52.16%) 333 (47.84%)	N/A
Entry vessel Cephalic vein Thoraco-acromial vein IJV Other^2^	593 (85.20%) 68 (9.77%) 33 (4.74%) 2 (0.29%)	594 (85.34%) 84 (12.07%) 18 (2.59%) 0 (0.00%)	0.0441				
				Post-operation quality Catheter-nut angle Tip location	169.86 ± 7.15 1.06 ± 1.30	169.87 ± 7.60 1.18 ± 1.45	0.9887 0.1059
Operation year 2012 2013 2014 2015 2016 2017 2018	199 (28.59%) 258 (37.07%) 44 (6.32%) 38 (5.46%) 117 (16.81%) 37 (5.32%) 3 (0.43%)	0 (0.00%) 37 (5.32%) 176 (25.29%) 157 (22.56%) 70 (10.06%) 139 (19.97%) 117 (16.81%)	<0.0001				
				Functional period (days) ^3^	667.90 ± 589.66	518.00 ± 452.81	<0.0001
				Follow-up status Alive Expired AAD	367 (52.73%) 191 (27.44%) 138 (19.83%)	482 (69.25%) 156 (22.41%) 58 (8.33%)	<0.0001

^1^ p-value was calculated by chi-square test or Fisher’s exact test.

^2^ Other: (EJV/axillary vein).

^3^ Functional period (days) defined as operation date to pre-intervention date, expiry date.

The total numbers of cases with complications in the silicone and polyurethane group are summarized in S2 Table. In total, 47 patients were identified with catheter related complications and total complication rate and incidence were 3.38% (47/1392), and 0.057 episodes per 1000 catheter days, respectively. We further subdivided both groups according to entry vessel in order to identify complication rate and incidence in the two groups, by entry vessel (Table 2). Only cephalic vein, thoraco-acromial vein and internal jugular vein were utilized for entry vessel after matching. The complication rates for silicone and PU catheters implanted via the cephalic vein are shown in Table 2, and clarify that there were no significant statistical differences in infection (2.02% vs.1.01%, p = 0.1531), malfunction (0.51% vs. 0.17%, p = 0.3740), migration (0.51% vs. 0.84%, p = 0.7256) and deep vein thrombosis (0.51% vs. 0.51%, p = 1.000) between the groups. The complication incidences for silicone and PU catheters implanted via the cephalic vein show there were no statistically significant differences in infection (0.03 vs. 0.02, p = 0.3952), malfunction (0.008 vs. 0.003, p = 0.4732), migration (0.008 vs. 0.016, p = 0.2862), and deep vein thrombosis (0.008 vs. 0.01, p = 0.7428). For catheters implanted via the thoraco-acromial and internal jugular vein, neither complication rate nor incidence showed any statistical difference between the silicone and polyurethane groups (Table 2).

**Table 2:** Comparison of complication rates and incidences between polyurethane and silicone tubes.

Complication rate							
Entry vessel Catheter type No. Complication	Cephalic vein			Thoraco-acromial vein			Total
	silicone	PU	p-value^1^	silicone	PU	p-value^1^	
	593	594		68	84		1392
Infection	2.02%	1.01%	0.1531	0	1.19%	1.0000	1.44%
Malfunction	0.51%	0.17%	0.3740	1.47%	1.19%	1.0000	0.57%
Migration	0.51%	0.84%	0.7256	1.47%	2.38%	1.0000	0.79%
Rotation	0	0	N/A	0	0	N/A	0.07%
Deep vein thrombosis	0.51%	0.51%	1.0000	1.47%	0	0.4474	0.50%
Entry vessel Catheter type No. Complication	Internal jugular vein			Other (EJV/axillary vein)			
	silicon	PU	p-value^1^	silicone	PU	p-value^1^	
	33	18		2	0		
Infection	0	5.56%	0.3529	0	0	N/A	
Malfunction	6.06%	0	0.5341	0	0	N/A	
Migration	0	0	N/A	0	0	N/A	
Rotation	3.03%	0	1.0000	0	0	N/A	
Deep vein thrombosis	0	0	N/A	0	0	N/A	
Complication incidence per 1000 catheter days							
Entry vessel Catheter type Days Complication	Cephalic vein			Thoraco-acromial vein			Total
	silicone	PU	p-value^2^	silicone	PU	p-value^2^	
	399335	305460		37433	44325		825385
Infection	0.030	0.020	0.3952	0	0.023	0.9964	0.024
Malfunction	0.008	0.003	0.4723	0.027	0.023	0.9049	0.010
Migration	0.008	0.016	0.2862	0.027	0.045	0.6687	0.013
Rotation	0	0	N/A	0	0	N/A	0.001
Deep vein thrombosis	0.008	0.010	0.7428	0.027	0	0.9963	0.008
Entry vessel Catheter type Days Complication	Internal jugular vein			Other (EJV/axillary vein)			
	silicon	PU	p-value2	silicone	PU	p-value2	
	24559	10743		3530	0		
Infection	0	0.093	0.9972	0	0	N/A	
Malfunction	0.081	0	0.9970	0	0	N/A	
Migration	0	0	N/A	0	0	N/A	
Rotation	0.041	0	0.9971	0	0	N/A	
Deep vein thrombosis	0	0	N/A	0	0	N/A	

^1^p-value was calculated by chi-square test or Fisher’s exact test.

^2^ p-value was calculated by Poisson regression.

Incidence rate = complication case/sum of person-days*1000.

**Fig 2:**
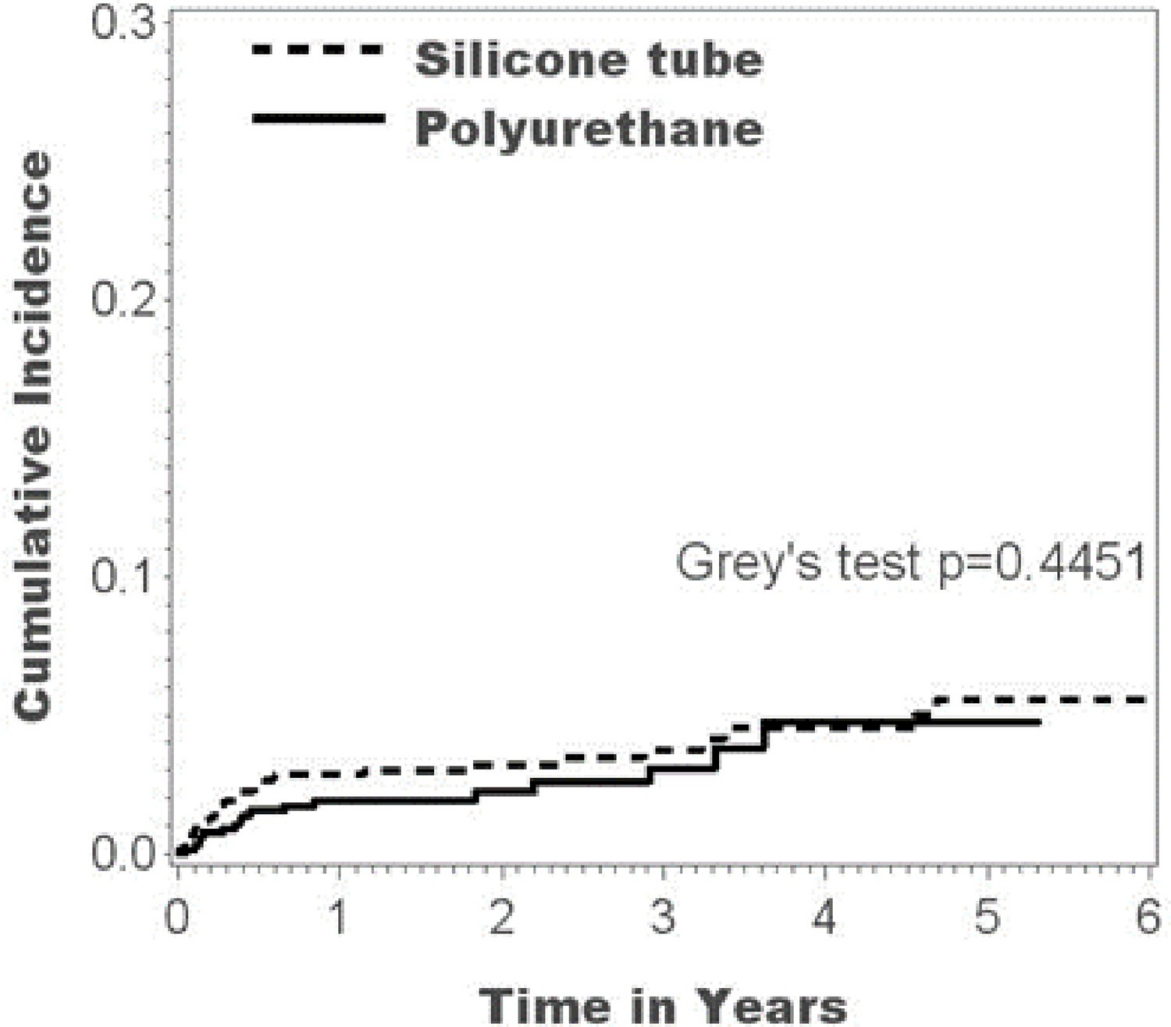
Cumulative incidence of complications.

From the view of functional period of implanted intravenous ports, not only catheter related complications but also disease progression would affect the long-term result of an implanted port. Therefore, we compared the cumulative complication incidence between the silicone group and the polyurethane group. The cumulative complication incidence in the silicone and polyurethane group showed no statistically significant difference (Fig 2, p = 0.4451).

## Discussion

From the point of view of embedded materials, the catheter is the only component that really resides within the vessel and plays a crucial role in catheter related complications which are of major concern for clinical practitioners. Two biocompatible materials, polyurethane and silicone are used in the manufacture of catheters. Both materials undergo degradation, which may lead to broken integrity [4–6]. Besides many other factors, including implantation method, quality of implantation and patients’ characteristics, catheter material may also affect the long-term outcome of an implanted port. In order to analyze the real performance of the catheter material, we tried to unify the heterogeneity between the polyurethane and silicone group, using a study design that differed completely from previous studies. First, we performed the implantation procedure and follow up using a standard algorithm and quantified implantation quality control measurements, including nut-catheter angle and tip location, in order to eliminate individual variations among surgeons [14,22]. In addition, we compared patients with similar body type using body mass index (BMI), and those undergoing similar treatment protocols for underlying malignancy in order to eliminate individual variations among patients. Furthermore, we followed a standard maintenance protocol with recommended minimal irrigation volume to minimize residual medication and protein deposit [24].

**Fig 3:**
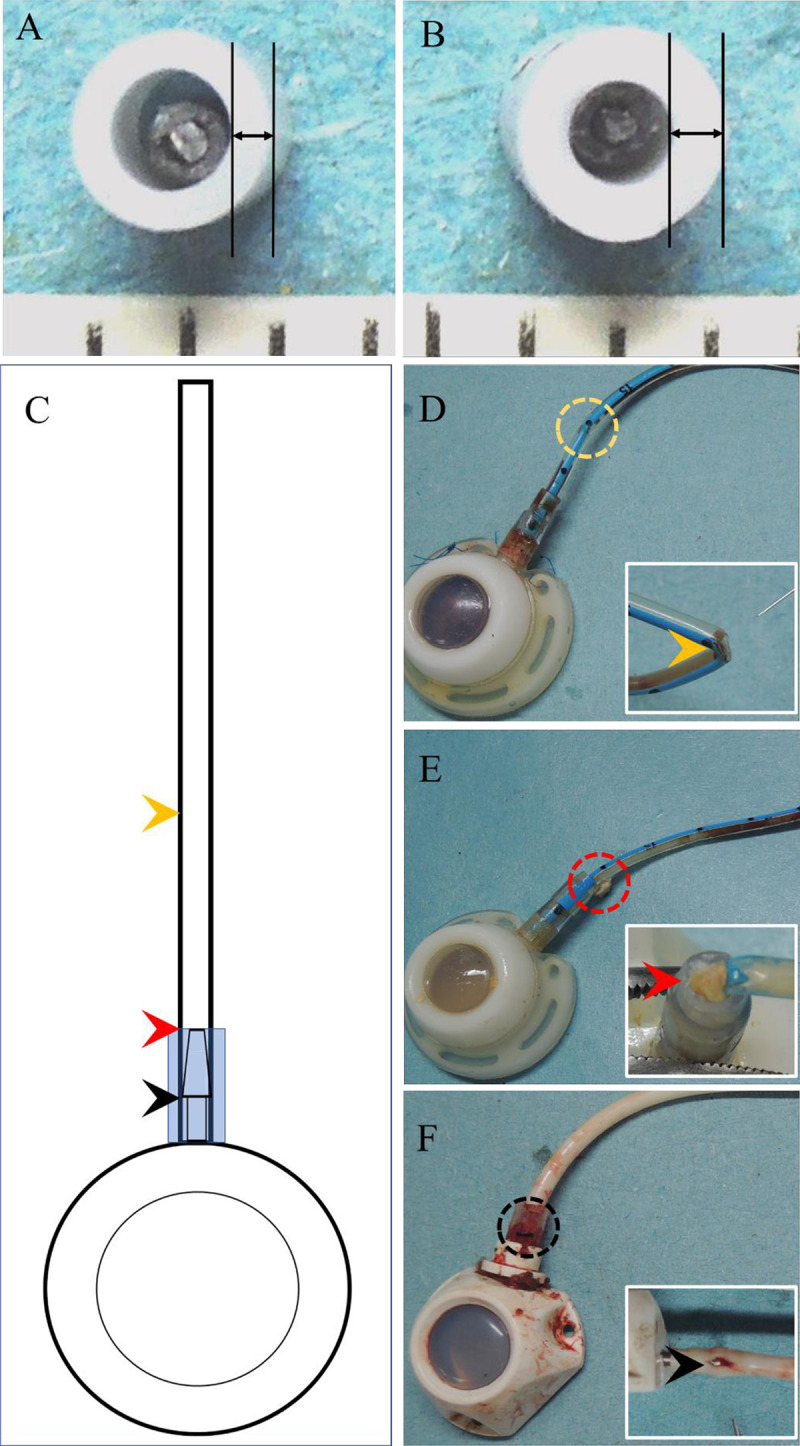
Structural difference among different materials and actual presentation of catheter fracture. A. Fr.6 Polyurethane catheter: Larger inner diameter and lesser wall thickness. More residual space after inserting a 0.035-inch Terumo wire during cannulation, which leads to less friction between wire and catheter. B. Fr.7 Silicone catheter: Smaller inner diameter and greater wall thickness. Small inner diameter after inserting a 0.035-inch Terumo wire during cannulation, which leads to more friction between wire and catheter. C. Three potential structure weakness points were identified during port revisions. D. Pinch-off syndrome related catheter fracture. It was caused by repetitive compressive forces exerted on the catheter result in catheter fracture related to material fatigue. E. Catheter fracture at junction site between locking nut and proximal catheter. It was caused by compressive forces that generated by surrounding soft tissue focused on unction site between locking nut and proximal catheter. F. Catheter stretched over the protruding stud of the connecting tube. Longitudinal fracture was identified at the point where the catheter overlies the protruding stud of the connecting tube.

After matching, the groups were completely corrected for patient age, gender, BMI, and underlying malignancy. Similar nut-catheter angle (169.86° ± 7.15° vs. 169.87° ± 7.60°, p = 0.9887) and tip locations (1.06 ± 1.30 cm vs. 1.18 ± 1.45cm, p = 0.1058) were identified (Table 1), suggesting similar implantation quality between the silicone and polyurethane groups. The standard algorithm assured a unified incision area based on a superficial landmark, ie, the coracoid process, and avoided unnecessary soft tissue dissection, resulting in reduced operation time in the vessel cutdown method (p< 0.0001). The endovascular technique meant more catheters were implanted via the thoraco-acromial vein, resulting in a change in choice of entry vessel (p = 0.0441) and operation method (p = 0.0095). Operation time for wire assisted approach may be related to inner caliber difference in catheters of different materials (p = 0.008). Polyurethane catheter has less wall thickness and larger intraluminal caliber (Fig 3A and 3B) that offers less resistance during cannulation. Since polyurethane ports only became available after the year 2013, this may be the basis for the difference in functional period (p < 0.0001) and follow up status (p<0.0001). Once surgeons had become familiar with the standard algorithm and endovascular techniques there was less variation in incision creation, and more challenging native vessels, such as those with small caliber and tortuous route, could be overcome by wire assisted techniques and used as entry vessels.

With regard to the cumulative complication incidence, there was no statistically significant difference between the two groups (Fig 2, p = 0.4451). Also, the complication rates and incidences were similar for the two groups except in the case of catheter fracture (Table 2); a finding that differed notably from previous studies [15–17]. This was because the standard algorithm that we followed has two crucial principles, namely, no more subclavian vein approach and creation of the embedding pocket over the pectoralis major fascia with adequate size. The former avoids the pinch-off syndrome caused by repetitive compression generated by the 1st rib and clavicle (Fig 3C and 3D) [18,19], and the latter avoids catheter impingement caused by persistent external forces generated by the surrounding tissue (Fig 3C and 3E) [19]. From the literature review, another potential fracture site is the mounting of the catheter at the protruding stud of the connecting tube [19,25]. At the protruding stud, the catheter material is stretched and the locking nut with its conjugate inner structure is locked onto the protruding stud of the connecting tube. The shear forces generated by the locking nut and protruding stud of the connecting tube are focused on the reduced wall thickness which may lead to broken integrity (Fig 3C and 3F) [19,25]. This is frequently seen after longer periods of implantation [26], however, we did not observe it in this study. Further studies are warranted to investigate the potential fracture site at the protruding stud of the connecting tube for possible clinical significance in determining ideal timing for tube removal.

Based on our findings, both silicone and polyurethane materials offer sufficient structural stability to maintain integrity. However, some limitations to this study remain. Despite the nature of this study being retrospective, the relatively large sample size after minimizing individual variations among surgeons and patients makes the cohort more homogenous and contributes to a more reliable conclusion. In addition, a limited number of patients (n = 7) suffered the complication of deep vein thrombosis, prohibiting further analysis of the relationship with the underlying malignancy. Furthermore, data on the microscopic presentation, such as surface roughness, elasticity, and degradation status of the implanted catheters were not available, thus limiting further analysis of the relationship between materials and complications. However, from a clinical point of view, both silicone and polyurethane catheter ports could serve as reliable vascular access for patients, in spite of their limitations.

## Conclusion

As long as external stress forces generated by surrounding structures and focused on potential weakness points can be avoided, both silicone and polyurethane materials offer sufficient structural stability for ports and catheters to serve as reliable vascular access for patients.

## Supporting information

S1 Table Descriptive data of ports with silicone and polyurethane catheters (before matching). (DOCX)

S2 Table Complication case numbers for polyurethane and silicone catheters. (DOCX)
