# Supplementary material for: Does catheter material affect functional performance of intravenous ports via the superior vena cava?
Source: PLoS One. 2021 Oct 27;16(10):e0253818. doi: 10.1371/journal.pone.0253818 (PMC8550392; doi:10.1371/journal.pone.0253818)
Supplement: S1 Table — (DOCX) [file pone.0253818.s001.docx]

S1 Table. Descriptive data of ports with silicone and polyurethane catheters (Before matching).

|  | Silicone  (n=1,226)  N(%)/ mean±SD | Polyurethane  (n=1,679)  N (%)/ mean±SD | p-value |  | Silicone  (n=1,226)  N(%)/mean± SD | Polyurethane  (n=1,679)  N (%)/ mean± SD | p-value |
| --- | --- | --- | --- | --- | --- | --- | --- |
| Gender  Female  Male | 483 (39.40%)  743 (60.60%) | 739 (44.01%)  940 (55.99%) | **0.0128** | Operation method  Vessel cutdown  Wire assistance without puncture  Wire assistance with puncture  Wire assistance with venogram  a. Over the wire  b. Modified puncture  Echo guide puncture | 772 (62.97%)  265 (21.62%)  138 (11.26%)  3 (0.24%)  6 (0.49%)  42 (3.43%) | 1,062 (63.25%)  332 (19.77%)  213 (12.69%)  9 (0.54%)  31 (1.85%)  32 (1.91%) | **0.0010** |
| Age | 58.69 ± 12.81 | 58.65 ± 12.88 | 0.9397 |  |  |  |  |
| BH | 161.25 ± 8.94 | 161.15 ± 8.86 | 0.7784 |  |  |  |  |
| BW | 60.75 ± 11.99 | 60.77 ± 12.05 | 0.9543 |  |  |  |  |
| BMI | 23.28 ± 3.83 | 23.34 ± 3.91 | 0.6382 |  |  |  |  |
| Malignancy  Head and neck  Thorax  Abdomen  Pelvis  Soft tissue  Hematology  Other | 159 (12.97%)  561 (45.76%)  383 (31.24%)  7 (0.57%)  12 (0.98%)  128 (10.44%)  8 (0.65%) | 139 (8.28%)  676 (40.26%)  749 (44.61%)  5 (0.30%)  5 (0.30%)  119 (7.09%)  13 (0.77%) | **<0.0001**  **0.0031**  **<0.0001**  0.2569  **0.0175**  **0.0014**  0.7021 |  |  |  |  |
|  |  |  |  | Operation time  Vessel cutdown  Wire assistance without puncture  Wire assistance with puncture  Wire assistance with venogram  a. Over the wire  b. Modified puncture  Echo guide puncture | 29.29 ± 9.88  32.99 ± 9.92  44.64 ± 15.90  27.67 ± 10.97  49.67 ± 15.54  61.00 ± 21.50 | 25.09 ± 9.97  27.49 ± 9.81  37.42 ± 15.09  34.89 ± 9.85  48.42 ± 16.91  60.81 ± 13.50 | <0.0001  <0.0001  <0.0001  0.3081  0.8681  0.9635 |
| Side  Right  Left | 1,106 (90.21%)  120 (9.79%) | 1,496 (89.10%)  183 (10.90%) | 0.3331 |  |  |  |  |
|  |  |  |  | Port type  B'Braun Fr. 6.5 (Silicone)  Polysite Fr.7 (Silicone)  Bard power port Fr.6 (Polyurethane)  Bard Fr.6/8 X port (Polyurethane) | 849 (69.25%)  377 (30.75%)  0 (0.00%)  0 (0.00%) | 0 (0.00%)  0 (0.00%)  927 (55.21%)  752 (44.79%) | NA* |
| Entry vessel  Cephalic vein  Thoracoacromial vein  IJV  Other | 1,052 (85.81%)  129 (10.52%)  43 (3.51%)  2 (0.16%) | 1,449 (86.30%)  197 (11.73%)  32 (1.91%)  1 (0.06%) | 0.0332 |  |  |  |  |
|  |  |  |  | Post-operation quality  Catheter-nut angle  Tip location | 169.97 ± 7.25  1.06 ± 1.28 | 169.73 ± 7.66  1.22 ± 1.57 | 0.4018  **0.0026** |
| Follow-up status  　Alive  Expire  AAD | 642 (52.37%)  355 (28.96%)  229 (18.68%) | 1,151 (68.55%)  394 (23.47%)  134 (7.98%) | <0.0001 |  |  |  |  |
|  |  |  |  | Functional period (day) | 612.07 ± 560.22 | 500.04 ± 441.71 | **<0.0001** |
|  |  |  |  |  |  |  |  |
|  |  |  |  |  |  |  |  |

^1^ p-value was calculated by chi-square test or Fisher’s exact test

^2^ Other: (EJV/axillary vein)

^3^ Function period (day) defined as operation date to pre-intervention date, expired date
