# Supplementary material for: Does catheter material affect functional performance of intravenous ports via the superior vena cava?
Source: PLoS One. 2021 Oct 27;16(10):e0253818. doi: 10.1371/journal.pone.0253818 (PMC8550392; doi:10.1371/journal.pone.0253818)
Supplement: S2 Table — (DOCX) [file pone.0253818.s002.docx]

S2 Table. Complication case numbers for polyurethane and silicone catheters.

| Entry vessel  Catheter type  Complication No. | Cephalic vein | | Thoracoacromial vein | | Internal jugular vein | | Other (EJV/axillary vein) | |  |
| --- | --- | --- | --- | --- | --- | --- | --- | --- | --- |
|  | silicone | polyurethane | silicone | polyurethane | silicone | polyurethane | silicone | polyurethane | Total |
|  | 593 | 594 | 68 | 84 | 33 | 18 | 2 | 0 | 1392 |
| Infection | 12 | 6 | 0 | 1 | 0 | 1 | 0 | 0 | 20 |
| Malfunction | 3 | 1 | 1 | 1 | 2 | 0 | 0 | 0 | 8 |
| Migration | 3 | 5 | 1 | 2 | 0 | 0 | 0 | 0 | 11 |
| Rotation | 0 | 0 | 0 | 0 | 1 | 0 | 0 | 0 | 1 |
| Deep vein thrombosis | 3 | 3 | 1 | 0 | 0 | 0 | 0 | 0 | 7 |
